# The impact of environmental variables on the spread of COVID-19 in the Republic of Korea

**DOI:** 10.1038/s41598-021-85493-y

**Published:** 2021-03-16

**Authors:** Yong Kwan Lim, Oh Joo Kweon, Hye Ryoun Kim, Tae-Hyoung Kim, Mi-Kyung Lee

**Affiliations:** 1 Department of Laboratory Medicine, Chung-Ang University College of Medicine, 102 Heukseok-ro, Dongjak-gu, Seoul, 06973 Republic of Korea; 2 Department of Urology, Chung-Ang University College of Medicine, Seoul, Republic of Korea

**Keywords:** Climate sciences, Environmental sciences, Diseases, Health care, Risk factors

## Abstract

Corona virus disease 2019 (COVID-19) has been declared a global pandemic and is a major public health concern worldwide. In this study, we aimed to determine the role of environmental factors, such as climate and air pollutants, in the transmission of COVID-19 in the Republic of Korea. We collected epidemiological and environmental data from two regions of the Republic of Korea, namely Seoul metropolitan region (SMR) and Daegu-Gyeongbuk region (DGR) from February 2020 to July 2020. The data was then analyzed to identify correlations between each environmental factor with confirmed daily COVID-19 cases. Among the various environmental parameters, the duration of sunshine and ozone level were found to positively correlate with COVID-19 cases in both regions. However, the association of temperature variables with COVID-19 transmission revealed contradictory results when comparing the data from SMR and DGR. Moreover, statistical bias may have arisen due to an extensive epidemiological investigation and altered socio-behaviors that occurred in response to a COVID-19 outbreak. Nevertheless, our results suggest that various environmental factors may play a role in COVID-19 transmission.

## Introduction

Since the identification of novel coronavirus (SARS-CoV-2) infection in Wuhan, China in December 2019, it has become one of the most significant global public health concerns^1^. As of January 31, 2020, a total of 102 million confirmed cases were reported worldwide with over 500,000 new cases of COVID-19 reported each day^2^. SARS-CoV-2 has similar, or slightly higher, reproductive capacity compared to the Middle East Respiratory Syndrome Coronavirus (MERS) or severe acute respiratory syndrome coronavirus 1 (SARS-CoV-1)^3^. Moreover, the rapid global spread of SARS-CoV-2 infection has been facilitated by its highly contagious nature. SARS-CoV-2 infection, also known as coronavirus disease 2019 (COVID-19), has also been reported to cause respiratory failure, multiorgan failure and even death in severe cases^4^.

Similar to that of other viral respiratory infections, the transmission of SARS-CoV-2 could also be affected by environmental factors including climate and air pollutants. Numerous studies have been performed to investigate the effect of meteorological factors on the transmission of SARS-CoV-2 in various countries^3,5–11^. Pani et al. reported that various factors including temperature, dew point, relative humidity, absolute humidity, and water vapor showed positive correlation with the transmission of SARS-CoV-2 in Singapore^3^. In Indonesia, only mean temperature was significantly correlated with the spread of COVID-19^11^. In South America, there was a highly significant correlation between absolute humidity and the spread of COVID-19^10^. These studies suggested that temperature and humidity seemed to favor the spread of the disease; however, the effect of climate factors on COVID-19 transmission remains unclear^12^.

Since the first confirmed case of COVID-19 in the Republic of Korea, on January 20, 2020, a total of 34,652 confirmed cases have been reported as of November 31, 2020, the majority of which were classified as domestic cases caused by community spread. The wide range of climate parameters experienced in the Republic of Korea, including variable temperature, rainfall, and relative humidity, make it a suitable location to investigate the environmental effects on SARS-CoV-2 transmission. Therefore, in this study, we aimed to investigate the role of environmental factors, such as climate and air pollutants in the transmission of SARS-CoV-2 by analyzing the relationship between these variables with the confirmed daily COVID-19 cases in two areas of the Republic of Korea, namely Seoul metropolitan region (SMR) and Daegu-Gyeongbuk region (DGR).

## Results

**Figure 1:**
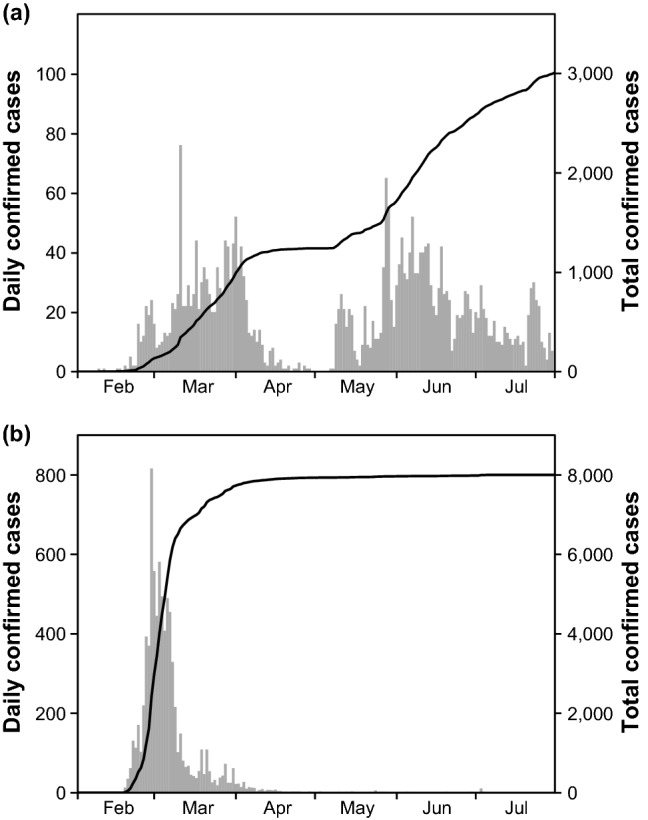
Daily confirmed cases (gray box) and total confirmed cumulative cases (solid line) of COVID-19 in Seoul metropolitan region (**a**) and Daegu-Gyeongbuk region (**b**).

During our study period, from February 2020 to July 2020, a total of 12,031 confirmed locally-transmitted COVID-19 cases were identified. Among these cases, 3010 (25.0%) and 7998 (66.5%) were from SMR and DGR, respectively. Figure 1 shows daily confirmed cases in SMR and DGR. An explosive outbreak was observed in DGR following religious gatherings between February and March. Meanwhile, there were several major cases of local cluster infections in SMR, and the number of confirmed cases has remained relatively constant in SMR since April.

Table S1 summarizes monthly variations in various environmental parameters in SMR and DGR. A large variation in temperature was noted between the months, with no significantly different meteorological parameters noted between SMR and DGR, except in July. The levels of atmospheric carbon monoxide, nitrogen dioxide, and sulfur trioxide were higher in SMR, and ozone was higher in DGR. However, significant differences were not observed in the PM_10_ and PM_2.5_ levels between the two regions, save for in March when both were significantly increased in SMR.

Table 1 summarizes the relationship between environmental factors and daily confirmed COVID-19 cases. Among the 16 environmental parameters, 11 and 10 were found to be significantly correlated with confirmed COVID-19 cases in SMR and DGR, respectively. However, the duration of sunshine and the ozone level were the only parameters determined to affect COVID-19 in both regions. Meanwhile, contradictory results were obtained regarding the relationship between cases and temperature variables and atmospheric pressure in the two regions. Specifically, all temperature related variables were positively correlated, and atmospheric pressure was inversely correlated with COVID-19 cases in SMR, while the opposite trend was observed in DGR.

**Table 1:** Summary of correlation results between COVID-19 and environmental parameters (n = 182) in SMR and DGR.

Parameters	SMR				DGR			
	Spearman rank correlation		Kendall rank correlation		Spearman rank correlation		Kendall rank correlation	
	r_S_	*p*-value	τ	*p*-value	r_S_	*p*-value	τ	*p*-value
Average temperature (°C)	0.507	< 0.001	0.350	< 0.001	− 0.152	0.026	− 0.179	< 0.001
Maximum temperature (°C)	0.459	< 0.001	0.312	< 0.001	− 0.212	0.002	− 0.209	< 0.001
Minimum temperature (°C)	0.524	< 0.001	0.371	< 0.001	− 0.112	0.104	− 0.135	0.007
Ground temperature (°C)	0.540	< 0.001	0.387	< 0.001	− 0.101	0.144	− 0.140	0.005
Diurnal temperature variation (°C)	0.115	0.094	0.081	0.089	0.306	< 0.001	0.228	< 0.001
Rainfall (mm)	0.074	0.279	0.056	0.290	− 0.174	0.011	− 0.138	0.012
Wind speed (m/s)	0.125	0.068	0.089	0.068	0.181	0.008	0.138	0.007
Relative humidity (%)	0.006	0.935	0.006	0.895	− 0.302	< 0.001	− 0.219	< 0.001
Atmospheric pressure (hPa)	− 0.460	< 0.001	− 0.320	< 0.001	0.136	0.048	0.123	0.015
**Duration of sunshine (h)**	**0.179**	**0.009**	**0.121**	**0.012**	**0.192**	**0.005**	**0.133**	**0.008**
Carbon monoxide (ppm)	− 0.330	< 0.001	− 0.245	< 0.001	− 0.152	0.026	− 0.105	0.06
Nitrogen dioxide (ppm)	− 0.251	< 0.001	− 0.172	< 0.001	0.008	0.91	0.014	0.787
**Ozone (ppm)**	**0.471**	** < 0.001**	**0.332**	** < 0.001**	**0.204**	**0.003**	**0.129**	**0.011**
Sulfur trioxide (ppm)	− 0.223	0.001	− 0.179	0.001	− 0.051	0.458	− 0.042	0.476
PM_10_ (μg/m^3^)	− 0.046	0.506	− 0.031	0.519	0.172	0.012	0.128	0.011
PM_2.5_ (μg/m^3^)	− 0.160	0.019	− 0.105	0.027	0.028	0.685	0.025	0.616

*SMR* Seoul metropolitan region, *DGR* Daegu-Gyeongbuk region.

Significantly correlated factors in both regions appear in bold font.

## Discussion

Numerous variables were included in the analysis for this study to clarify the relationship between the environment and confirmed COVID-19 cases. We found that the duration of sunshine and the ozone level were positively correlated with the spread of SARS-CoV-2 in the two selected regions, which contradicts the results of Ratnesar-Shumate et al. who reported that sunlight rapidly inactivates SARS-CoV-2 on surfaces^13^. We postulate that one potential explanation for our results is the increased risk of SARS-CoV-2 exposure associated with outdoor activities during days with a longer duration of sunlight^14^, resulting in increased transmission prior to inactivation of the virus by sunlight. Additionally, Daniele et al. reported that atmospheric pollution may influence the SARS-CoV-2 outbreak in Italy^15^. We, therefore, also analyzed the effect of air pollutants on the spread of SARS-CoV-2 and found no correlation between SARS-CoV-2 transmission and air pollutants, with the exception of ozone, suggesting that air pollutants do not represent a significant environmental driver of SARS-CoV-2 transmission in the Republic of Korea. The ozone level significantly affected the transmission of SARS-CoV-2, and we hypothesize that the ozone level would disrupt the respiratory epithelial barrier^16,17^, making the public more susceptible to the SARS-CoV-2 infection.

Numerous studies have also reported a strong association between COVID-19 transmission and temperature^3,5,11,18^. We, therefore, selected two regions that showed similar temperature fluctuations during the study period; however, contradictory results were observed for all temperature variables between the two regions. Specifically, SARS-CoV-2 transmission increased as the temperature increased in SMR; whereas, we observed an increase in the number of cases with a decrease in temperature in DGR. The large outbreak that occurred following religious events in DGR between February and March 2020 may have contributed to these conflicting results. Moreover, in response to these outbreaks, an extensive epidemiological investigation was performed for members who participated in this event and for their close immediate contacts. Moreover, after this outbreak between February and March, strict measures were set in place to ensure proper maintenance of personal hygiene, including wearing of masks, use of hand sanitizers, and social distancing^19^, resulting in only a few cases of local clustering and sporadic infections reported in SMR and DGR. We could, therefore, assume that changes in socio-behavioral factors in response to the outbreaks had a greater impact than the environmental factors on the spread of SARS-CoV-2. However, extensive and rapid epidemiological investigations would also lead to an increase in the number of confirmed cases within a short period of time, which would subsequently skew the statistical results related to the effects of temperature variables. Therefore, one should consider that a large number of confirmed cases identified in these types of situations would greatly influence the correlation analysis between environmental factors and disease transmission, resulting in false negative or positive results.

Despite the significant results demonstrating the effect of environmental factors on COVID-19, there were several limitations noted in our study. First, the classification of locally infected cases and imported cases may have been affected by the extensive epidemiological investigation. Therefore, the number of confirmed cases due to community infection may be inaccurate, although we collected the epidemiological data from Korea Centers for Disease Control and Prevention (KCDC). Second, in the early days of the COVID-19 pandemic, there was a shortage of molecular tests for COVID-19 diagnosis, therefore, the impact of the environmental factors might not be accurately reflected. Third, we could not consider individual health factors, such as personal hygiene or exposure risk to SARS-CoV-2, in the statistical analyses.

The environment plays a significant role in the regional spread of COVID-19, and our results demonstrated that the duration of sunshine and ozone level were positively correlated with SARS-CoV-2 transmission. However, the number of confirmed COVID-19 cases may have also been impacted by various additional factors, including the completion of a large epidemiological investigation within the study period or changing socio-behaviors based on cluster outbreaks. Hence, these factors could create a bias in the statistical results. For these reasons, further studies should be conducted to confirm the relationship between environmental factors and SARS-CoV-2 transmission with sufficient epidemiological data generated over an extended period of time.

## Methods

### Study area and confirmed COVID-19 cases

The Republic of Korea lies between latitudes 33° and 39° N, and longitudes 124° and 130° E and tends to have a humid continental and subtropical climate with typically large seasonal temperature fluctuations. We selected the SMR and DGR areas that had relatively higher numbers of COVID-19 cases. The data for daily confirmed cases of COVID-19 from February 1, 2020 to July 31, 2020 were obtained from the KCDC website (http://www.cdc.go.kr). Our study aimed to exclusively investigate the effect of environmental factors on COVID-19 transmission, therefore, we have only included the confirmed cases caused due to communal spread.

### Meteorological and air pollution data

The meteorological data were collected from the database of Korea Meteorological Administration (KMA; https://data.kma.go.kr/). The KMA provides high-resolution meteorological information for specific areas on a flexible web-based platform^20,21^. Additionally, we collected the daily records on ten basic meteorological factors, including average temperature, maximum temperature, minimum temperature, ground temperature, diurnal temperature variation, amount of rainfall, wind speed, relative humidity, atmospheric pressure, and duration of sunshine. Information on air pollution across the country was collected from AIRKOREA (http://airkorea.or.kr), which was launched by the Korean Ministry of Environment and is a database that provides an account of daily air pollutant levels including carbon monoxide, nitrogen dioxide, ozone, sulfur trioxide, PM_10_, and PM_2.5_.

### Ethical statement

Data on environmental conditions and COVID-19 confirmed cases were obtained from official reports provided in a public database, therefore ethical review was not required.

### Statistics

Datasets were analyzed using R version 3.6.3 (http://www.R-project.org/). Descriptive analyses for monthly meteorological factors and air pollutants during the study period were performed for both regions (SMR, DGR). Student's *t*-test was used to determine the statistical difference in monthly environmental variables between the regions. *P*-values < 0.05 were considered significant. To examine the relationship between environmental factors and daily COVID-19 confirmed cases, we conducted Spearman rank correlation test and Kendall rank correlation test. Factors were considered to affect SARS-CoV-2 if significant differences were observed in both statistical tests.

## Supplementary Information

Supplementary Information.

## Data Availability

The data analyzed in this study are available on GitHub at https://github.com/yoorer/COVID_ENV.

## Code availability

Codes are available on GitHub at https://github.com/yoorer/COVID_ENV.
