# Supplementary material for: The impact of environmental variables on the spread of COVID-19 in the Republic of Korea
Source: Sci Rep. 2021 Mar 16;11:5977. doi: 10.1038/s41598-021-85493-y (PMC7966806; doi:10.1038/s41598-021-85493-y)
Supplement: Supplementary file 1 — Supplementary Information. [file 41598_2021_85493_MOESM1_ESM.docx]

**Supplementary Information**

**The impact of environmental variables on the spread of COVID-19 in the Republic of Korea**

Yong Kwan Lim, M.D.^1^, Oh Joo Kweon, M.D.^1^, Hye Ryoun Kim, M.D.^1^, Tae-Hyoung Kim, M.D.^2^, Mi-Kyung Lee, M.D.^1,*^

^1^Department of Laboratory Medicine, Chung-Ang University College of Medicine, Seoul, Republic of Korea

^2^Department of Urology, Chung-Ang University College of Medicine, Seoul, Republic of Korea

*Corresponding author, email: cpworld@cau.ac.kr

**Table S1.** Monthly COVID-19 infection and environmental parameters (mean ± SD) in SMR and DGR

|  | February | | |  | March | | |  | April | | |
| --- | --- | --- | --- | --- | --- | --- | --- | --- | --- | --- | --- |
| Parameters | SMR | DGR | *p*-value |  | SMR | DGR | *p*-value |  | SMR | DGR | *p*-value |
| Total cases (*n*) | 119 | 2,428 |  |  | 824 | 5,282 |  |  | 299 | 220 |  |
| Average temperature (°C) | 1.9 ± 4.4 | 3.5 ± 3.5 | 0.117 |  | 7.0 ± 3.3 | 8.1 ± 2.8 | 0.171 |  | 10.4 ± 2.3 | 11.2 ± 2.5 | 0.225 |
| Maximum temperature (°C) | -2.7 ± 4.7 | -1.7 ± 3.9 | 0.381 |  | 0.9 ± 2.9 | 1.5 ± 2.6 | 0.387 |  | 4.3 ± 2.3 | 4.3 ± 2.5 | 0.961 |
| Minimum temperature (°C) | 7.1 ± 4.8 | 9.4 ± 4.1 | 0.062 |  | 13.2 ± 4.1 | 14.7 ± 3.8 | 0.132 |  | 16.6 ± 3.2 | 18.1 ± 3.4 | 0.101 |
| Ground temperature (°C) | 2.8 ± 2.8 | 4.3 ± 2.8 | 0.047 |  | 7.9 ± 3.3 | 10.2 ± 3.3 | 0.009 |  | 13.9 ± 2.5 | 15.3 ± 2.7 | 0.038 |
| Diurnal temperature variation (°C) | 9.8 ± 3.1 | 11.1 ± 3.6 | 0.157 |  | 12.3 ± 3.3 | 13.2 ± 3.6 | 0.295 |  | 12.4 ± 3.2 | 13.7 ± 3.9 | 0.136 |
| Rainfall (mm) | 1.8 ± 4.3 | 2.0 ± 7.1 | 0.920 |  | 0.5 ± 1.7 | 0.6 ± 2 | 0.764 |  | 0.6 ± 2.1 | 1.2 ± 4.1 | 0.444 |
| Wind speed (m/s) | 1.8 ± 0.8 | 2.1 ± 1.0 | 0.129 |  | 2.2 ± 0.9 | 2.3 ± 0.9 | 0.950 |  | 2.6 ± 0.7 | 2.6 ± 0.7 | 0.998 |
| Relative humidity (%) | 69.8 ± 12.1 | 63.5 ± 16.1 | 0.095 |  | 57.5 ± 11.4 | 59.6 ± 14.0 | 0.534 |  | 56.9 ± 12.4 | 53.0 ± 14.5 | 0.268 |
| Atmospheric pressure (hPa) | 1017.2 ± 5.1 | 1010.2 ± 5.1 | <0.001 |  | 1010.2 ± 4.7 | 1001.3 ± 13.0 | <0.001 |  | 1009.6 ± 3.5 | 1003.1 ± 3.5 | <0.001 |
| Duration of sunshine (hr) | 6.2 ± 3.3 | 6.6 ± 3.2 | 0.636 |  | 8.2 ± 3.2 | 7.5 ± 3.7 | 0.460 |  | 9.6 ± 3.2 | 8.8 ± 3.5 | 0.371 |
| Carbon monoxide (ppm) | 0.58 ± 0.14 | 0.53 ± 0.14 | 0.206 |  | 0.49 ± 0.09 | 0.42 ± 0.06 | <0.001 |  | 0.42 ± 0.05 | 0.39 ± 0.05 | 0.016 |
| Nitrogen dioxide (ppm) | 0.0286 ± 0.0116 | 0.0185 ± 0.0069 | <0.001 |  | 0.0244 ± 0.0104 | 0.0148 ± 0.005 | <0.001 |  | 0.0182 ± 0.006 | 0.0115 ± 0.0036 | <0.001 |
| Ozone (ppm) | 0.0207 ± 0.0073 | 0.0264 ± 0.0069 | 0.003 |  | 0.0295 ± 0.0062 | 0.0334 ± 0.0071 | 0.026 |  | 0.0389 ± 0.0058 | 0.043 ± 0.0082 | 0.028 |
| Sulfur trioxide (ppm) | 0.0036 ± 0.0006 | 0.0034 ± 0.0006 | 0.383 |  | 0.0035 ± 0.0005 | 0.0032 ± 0.0005 | 0.011 |  | 0.0032 ± 0.0005 | 0.0029 ± 0.0005 | 0.023 |
| PM_10_ (㎍/㎥) | 40.4 ± 18.5 | 36.7 ± 16.6 | 0.415 |  | 45.2 ± 9.7 | 37.1 ± 9.4 | 0.002 |  | 43.2 ± 14.6 | 38.3 ± 14.1 | 0.190 |
| PM_2.5_ (㎍/㎥) | 26.5 ± 14.3 | 23.9 ± 12.9 | 0.473 |  | 23.9 ± 10.4 | 19.4 ± 6.2 | 0.038 |  | 19.1 ± 6.8 | 17.6 ± 6.2 | 0.388 |

**Table S1.** Continued

|  | May | | |  | June | | |  | July | | |
| --- | --- | --- | --- | --- | --- | --- | --- | --- | --- | --- | --- |
| Parameters | SMR | DGR | *p*-value |  | SMR | DGR | *p*-value |  | SMR | DGR | *p*-value |
| Total case (*n*) | 448 | 34 |  |  | 875 | 18 |  |  | 445 | 16 |  |
| Average temperature (°C) | 17.2 ± 2.3 | 17.8 ± 2.3 | 0.299 |  | 22.7 ± 2 | 22.9 ± 1.7 | 0.757 |  | 23.2 ± 1.6 | 22 ± 1.9 | 0.006 |
| Maximum temperature (°C) | 12.5 ± 2.3 | 12.2 ± 2.6 | 0.566 |  | 18.4 ± 1.6 | 17.6 ± 1.8 | 0.108 |  | 20 ± 1.5 | 19 ± 1.9 | 0.025 |
| Minimum temperature (°C) | 22.8 ± 3.3 | 23.9 ± 3.4 | 0.202 |  | 28 ± 3.0 | 28.7 ± 2.8 | 0.333 |  | 27.3 ± 2.4 | 25.8 ± 3.0 | 0.039 |
| Ground temperature (°C) | 20.3 ± 2.9 | 22.7 ± 3.1 | 0.003 |  | 27.7 ± 3.7 | 28.4 ± 3.1 | 0.408 |  | 25.7 ± 2.5 | 25 ± 2.7 | 0.288 |
| Diurnal temperature variation (°C) | 10.3 ± 3.2 | 11.7 ± 3.8 | 0.116 |  | 9.6 ± 2.6 | 11 ± 3.2 | 0.064 |  | 7.3 ± 2.4 | 6.8 ± 3.0 | 0.522 |
| Rainfall (mm) | 3.7 ± 8.8 | 2.4 ± 6.4 | 0.491 |  | 3.9 ± 10.4 | 5.1 ± 10.1 | 0.669 |  | 9.1 ± 19.5 | 13.3 ± 21.1 | 0.419 |
| Wind speed (m/s) | 1.8 ± 0.4 | 1.9 ± 0.5 | 0.192 |  | 1.8 ± 0.5 | 1.7 ± 0.4 | 0.513 |  | 1.7 ± 0.6 | 1.4 ± 0.3 | 0.015 |
| Relative humidity (%) | 74.4 ± 14.5 | 69.6 ± 12.7 | 0.168 |  | 75.3 ± 8.3 | 71.6 ± 11.1 | 0.148 |  | 82.5 ± 7.8 | 86.8 ± 6.7 | 0.023 |
| Atmospheric pressure (hPa) | 1002.9 ± 4.5 | 997.4 ± 4.8 | <0.001 |  | 999 ± 4 | 993.6 ± 4.2 | <0.001 |  | 999.7 ± 3.6 | 992.4 ± 12.2 | 0.003 |
| Duration of sunshine (hr) | 6.1 ± 3.9 | 7.5 ± 3.8 | 0.139 |  | 7.2 ± 4.3 | 7.6 ± 4.2 | 0.724 |  | 4.6 ± 3.7 | 3.3 ± 3.7 | 0.184 |
| Carbon monoxide (ppm) | 0.42 ± 0.06 | 0.36 ± 0.05 | <0.001 |  | 0.43 ± 0.06 | 0.37 ± 0.05 | <0.001 |  | 0.4 ± 0.07 | 0.37 ± 0.08 | 0.090 |
| Nitrogen dioxide (ppm) | 0.0175 ± 0.0052 | 0.0097 ± 0.0022 | <0.001 |  | 0.0168 ± 0.0053 | 0.0108 ± 0.0026 | <0.001 |  | 0.0141 ± 0.0039 | 0.0097 ± 0.0023 | <0.001 |
| Ozone (ppm) | 0.0374 ± 0.0076 | 0.0420 ± 0.0078 | 0.020 |  | 0.0450 ± 0.0107 | 0.0461 ± 0.0089 | 0.657 |  | 0.0319 ± 0.0108 | 0.0284 ± 0.0085 | 0.157 |
| Sulfur trioxide (ppm) | 0.0031 ± 0.0004 | 0.0028 ± 0.0004 | 0.023 |  | 0.0031 ± 0.0004 | 0.0029 ± 0.0005 | 0.097 |  | 0.0028 ± 0.0003 | 0.0025 ± 0.0003 | <0.001 |
| PM_10_ (㎍/㎥) | 34.4 ± 17.1 | 35.6 ± 16.9 | 0.775 |  | 36.1 ± 12.2 | 33.5 ± 15.8 | 0.469 |  | 22.2 ± 11.6 | 19.3 ± 7.5 | 0.238 |
| PM_2.5_ (㎍/㎥) | 17.6 ± 7.1 | 17.6 ± 6 | 0.990 |  | 19.6 ± 6.9 | 17.9 ± 7.2 | 0.360 |  | 13.2 ± 8.2 | 11.4 ± 5.8 | 0.311 |

SMR, Seoul metropolitan region; DGR, Daegu-Gyeongbuk region
